# Identification of Hub Genes and Physiological Effects of Overexpressing the Photosynthesis-Related Gene *Soly720* in Tomato under High-CO_2_ Conditions

**DOI:** 10.3390/ijms25020757

**Published:** 2024-01-07

**Authors:** Shaowen Zheng, Lingbo Yang, Hao Zheng, Jiayue Wu, Zijian Zhou, Jieyun Tian

**Affiliations:** Horticulture College, Shanxi Agricultural University, Jinzhong 030801, China; zhsw4836@sxau.edu.cn (S.Z.); s20212216@stu.sxau.edu.cn (L.Y.); z20213240@stu.sxau.edu.cn (H.Z.); z20213577@stu.sxau.edu.cn (J.W.); z20213215@stu.sxau.edu.cn (Z.Z.)

**Keywords:** CO_2_ enrichment, greenhouse tomato, photosynthetic gene, WGCNA

## Abstract

Changes in the atmospheric CO_2_ concentration influence plant growth and development by affecting the morphological structure and photosynthetic performance. Despite evidence for the macro-effects of elevated CO_2_ concentrations on plant morphology and yield in tomato, the gene regulatory network and key genes related to cross-regulation have not been reported. To identify the hub genes and metabolic pathways involved in the response of tomato to CO_2_ enrichment, weighted gene co-expression network analysis was conducted using gene expression profiles obtained by RNA sequencing. The role of the photosynthesis-related gene *Soly720* (*Solyc01g007720*) in CO_2_-enriched tomato plants was explored. Tomato plants responded to CO_2_ enrichment primarily through RNA-related pathways and the metabolism of amino acids, fatty acids, and carbohydrates. The hub genes in co-expression networks were associated with plant growth and development, including cellular components and photosynthesis. Compared to wild-type plants, transgenic plants overexpressing the *Soly720* gene exhibited 13.4%, 5.5%, 8.9%, and 4.1% increases in plant height, stem diameter, leaf length, and leaf width, respectively, under high-CO_2_ conditions. The morphological improvements in transgenic plants were accompanied by enhancement of photosynthetic performance in terms of chlorophyll contents, photosynthetic characteristics, and key enzyme activities. This study elucidates the response network of tomato to CO_2_ enrichment and demonstrates the regulatory role of *Soly720* in photosynthesis under high-CO_2_ conditions.

## 1. Introduction

The carbon cycle between Earth’s atmosphere, land surface, and water constitutes a dynamically balanced global carbon exchange system. Terrestrial plants have been regarded as a fixation and absorption pool of carbon dioxide (CO_2_). For crop plants, the elevation of CO_2_ concentration in the environment is equivalent to gas fertilizer application, which can enhance plant biomass accumulation and yield formation [1]. When atmospheric CO_2_ concentrations are progressively exceeded, the CO_2_ absorption capacity of plants is compromised and limited. In this case, it is crucial to understand the adaptability of plants to rapidly changing climatic conditions. Therefore, substantial research has been conducted to characterize the response of CO_2_-enriched plants and the effects of CO_2_ enrichment on plant growth, morphology, and physiology [2,3].

Tomato (*Solanum lycopersicum* L., Solanaceae) is one of the most popular vegetable crops cultivated in greenhouses, especially across north and south China. Tomato fruit is rich in nutrients (e.g., vitamin C, sugars, proteins, organic acids, minerals) and bioactive substances (e.g., carotenoids, flavonoids, phenolic acids), which can impart health benefits to the human body [4,5]. Tomato provides a model plant for the study of physiological and biochemical mechanisms in Solanaceae vegetables. The completion of tomato genome sequencing and accumulation of gene annotation information have offered opportunities for exploring photosynthesis and carbohydrate metabolism in tomato and other Solanaceae vegetables [6].

It has been found that applying CO_2_ to tomato plants in the low-temperature season leads to increased plant growth rate, fruit setting rate, and yield [7]. At high temperatures, elevated CO_2_ concentrations contribute to plant photosynthesis by reducing the functional constraints on energy flux, electron transport, and redox homeostasis in tomato leaves [8]. As CO_2_ is the direct substrate of plant photosynthesis, changes in the CO_2_ concentration inevitably affect the photosynthetic performance of crops, which in turn influences their vegetative and reproductive growth. CO_2_ enrichment plays a positive role in leaf net photosynthesis, plant biomass accumulation, and crop quality improvement. At the flowering stage, dry matter accumulation in tomato roots, stems, and leaves is promoted under CO_2_ enrichment [9]. CO_2_ enrichment also affects the accumulation and distribution of carbohydrates in tomato and other plants [10,11], with enhanced activity of photosynthetic enzymes, such as ribulose-1,5-bisphosphate carboxylase/oxygenase (RuBisCO) and sedoheptulose-1,7-bisphosphatase (SBPase) [12,13].

In addition to direct response, there are other pathways through which cross-regulation and -response occur in plants under high-CO_2_ conditions. For example, the melatonin content in tomato leaves is increased in a high-CO_2_ environment, and endogenous melatonin levels affect the accumulation of sugars and starches [14]. Abscisic acid-deficient mutant plants show lower leaf stomatal conductance in a high-CO_2_ environment than in normal conditions [15]. At the molecular level, high CO_2_ concentrations affect gene expression in grape, maize, and rice [16,17,18]. Co-overexpression of two chloroplast glyceraldehyde-3-phosphate dehydrogenase (GAPDH) genes, *GAPA* and *GAPB*, in rice enhances the activity of GAPDH, leading to increased CO_2_ assimilation rate in transgenic plants under high-CO_2_ conditions [19]. Silencing of the chloroplast vesiculation gene in rice enables plants to maintain photorespiration under elevated CO_2_ conditions [20]. Therefore, chloroplast-related genes play vital roles in regulating plant response to CO_2_ enrichment.

In our previous study, high expression of the chloroplast-related gene *Soly720* (*Solyc01g007720*, Log_2_FC = 4.24) was observed in tomato plants under high-CO_2_ conditions [21]. However, little is known about the regulatory role of *Soly720* and whether there are other cross-regulatory networks and pathways in CO_2_-enriched tomato plants. In this study, the cross-response mechanisms of tomato to CO_2_ enrichment were explored by constructing the weighted gene co-expression networks. Through the analysis of gene expression patterns in modules with high relevance in the co-expression network, key genes and metabolic pathways were identified in response to CO_2_ enrichment in tomato. Furthermore, through a comprehensive analysis of transgenic plants, the effects of overexpressing *Soly720* on tomato plants were revealed based on morphological, photosynthetic, and enzymatical observations. The molecular mechanisms of tomato plant response to CO_2_ enrichment were deciphered, which could be helpful for breeding new crop varieties suitable for cultivation in high-CO_2_ environments.

## 2. Results

### 2.1. Construction of Weighted Gene Co-Expression Networks under CO_2_ Enrichment

**Figure 1:**
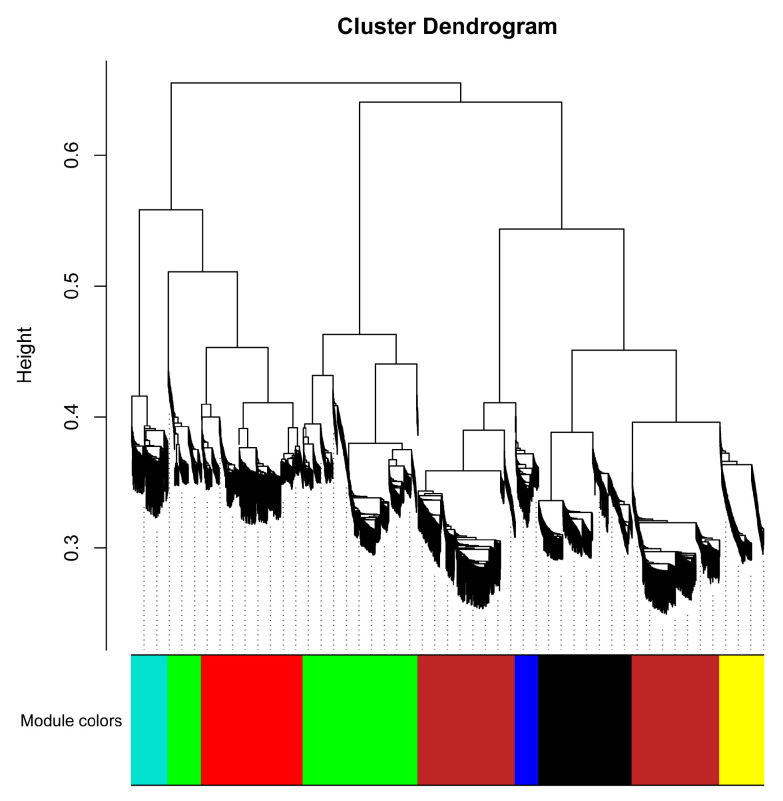
Cluster dendrogram from the weighted co-expression gene network analysis (WGCNA) of tomato plants under natural and high-CO_2_ conditions. Different colors represent various network modules based on their expression patterns.

**Figure 2:**
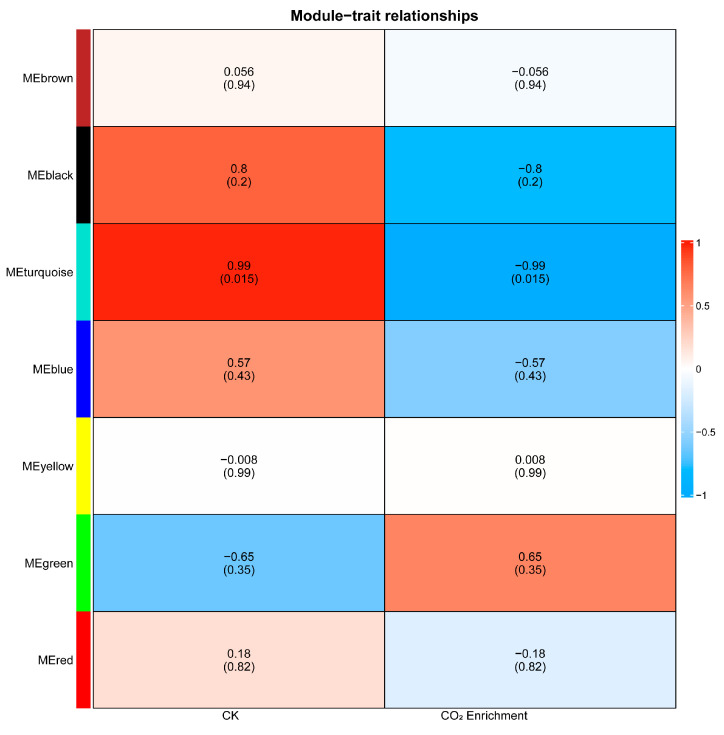
Correlations between network modules and CO_2_ concentrations in tomato plants. Data represent the correlation coefficients, with corresponding *p* values in brackets. The color gradient indicates the strength of the correlation.

To investigate the gene regulatory network of tomato under high-CO_2_ conditions, weighted gene co-expression network analysis (WGCNA) was conducted to identify modules associated with CO_2_ enrichment. The input genes were divided into seven modules based on their expression patterns and labeled with different colors: MEbrown, MEblack, MEturquoise, MEblue, MEyellow, MEgreen, and MEred (Figure 1). Among them, MEblack (normal control: *r* = 0.80; CO_2_ enrichment: *r* = −0.80) and MEturquoise (normal control: *r* = 0.99; CO_2_ enrichment: *r* = −0.99) showed significant positive or negative correlation with CO_2_ concentration. This suggests that members of the MEblack and MEturquoise modules were well representative of genes in response to CO_2_ enrichment (Figure 2).

**Figure 3:**
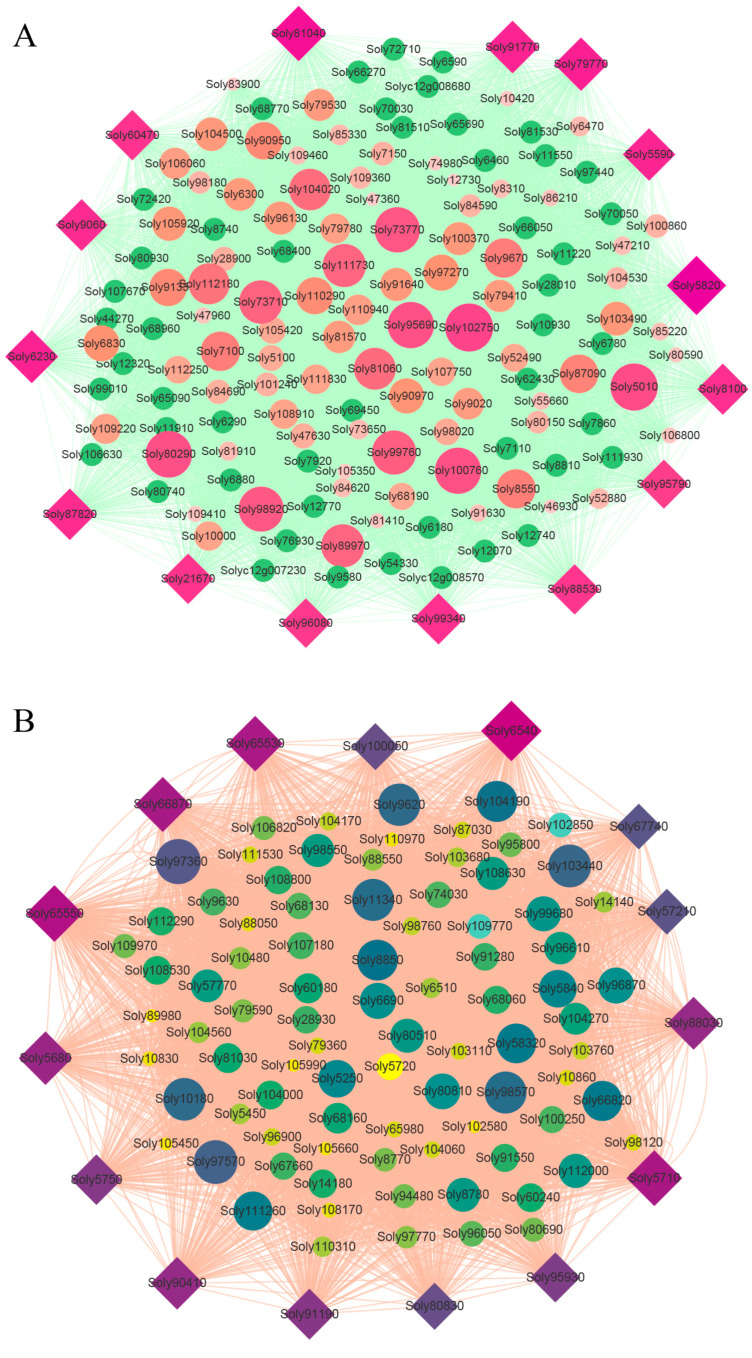
Co-expression network of the top 100 hub genes from the (**A**) MEblack and (**B**) MEturquoise modules. The size and color of nodes represent the connectivity of genes, and the diamonds represent the top 15 hub genes in the module.

The genes in the MEblack and MEturquoise modules were selected for further analysis. To visualize the interactions between the genes and metabolic pathways associated with CO_2_ enrichment, co-expression networks were constructed based on gene connectivity. In the MEblack module, the top 15 genes in terms of connectivity were *Soly5820*, *Soly81040*, *Soly79770*, *Soly6230*, *Soly91770*, *Soly5590*, *Soly9060*, *Soly87820*, *Soly8100*, *Soly99340*, *Soly60470*, *Soly21670*, *Soly88530*, *Soly96080*, and *Soly95790* (Figure 3A). Among them, the *Soly5820* gene had the highest connectivity with other genes and participated in organic cyclic compound binding (GO: 0097159). The *Soly6230* gene encoded cysteine protease and participated in cysteine-type peptidase activity (GO:0008234). *Soly81040*, *Soly91770*, and *Soly99340* were identified to be genes encoding RING-H2 finger proteins.

In the MEturquoise module, the top 15 hub genes were *Soly6540*, *Soly65550*, *Soly5710*, *Soly65530*, *Soly66870*, *Soly5680*, *Soly88030*, *Soly90410*, *Soly91190*, *Soly5750*, *Soly95930*, *Soly100050*, *Soly80830*, *Soly57210*, and *Soly67740* (Figure 3B). Among them, the *Soly6540* (*LOX2*.1) gene had the highest connectivity with other genes and was involved in encoding chloroplastic linoleate 13S-lipoxygenase 2-1. *Soly91190* encoded chloroplastic 3-phosphoshikimate 1-carboxyvinyltransferase, which participated in the function of cellular component: chloroplast (GO:0009507). *Soly5750* encoded chloroplastic linoleate 13S-lipoxygenase 2-1, which played a role in cellular component: chloroplast thylakoid (GO:0009534) and chloroplast stroma (GO:0009570). *Soly65530* took part in cell growth (GO:0016049), whereas *Soly66870* and *Soly57210* were involved in signal transduction mechanisms.

### 2.2. Comprehensive Analysis of Hub Genes in Co-Expression Networks

**Figure 4:**
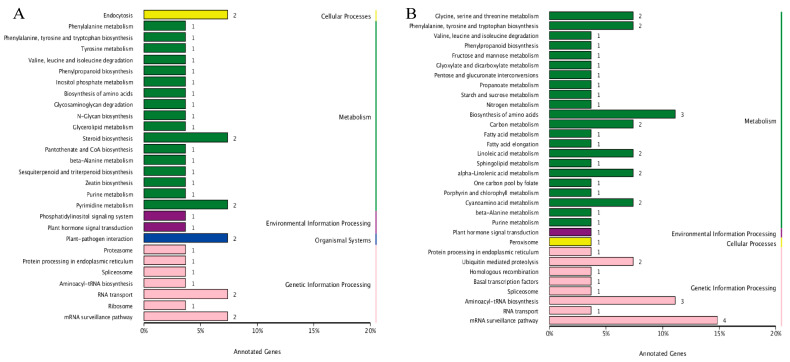
Functional annotation and Kyoto Encyclopedia of Genes and Genomes (KEGG) pathway enrichment of hub genes in the (**A**) MEblack and (**B**) MEturquoise modules.

To further investigate gene functions in the networks, functional annotation and functional enrichment analysis of the hub genes were performed based on the Gene Ontology (GO) and Kyoto Encyclopedia of Genes and Genomes (KEGG) databases. In the MEblack module (Figure 4A, Supplemental Table S1), two genes were enriched in endocytosis (ko04144: *Soly100860*, *Soly84690*), steroid biosynthesis (ko00100: *Soly110290*, *Soly111830*), pyrimidine metabolism (ko00240: *Soly89970*, *Soly47630*), and plant–pathogen interaction (ko04626: *Soly104530*, *Soly112250*). A total of four genes were enriched in RNA-related pathways: *Soly98180* and *Soly6470* in RNA transport (ko03013), and *Soly73650* and *Soly47360* in mRNA surveillance (ko03015).

Six genes in the MEblack module were enriched in amino acid metabolism-related pathways: *Soly47630* in beta-alanine metabolism (ko00410), *Soly28900* in valine, leucine, and isoleucine degradation (ko00280), *Soly6300* in phenylalanine metabolism (ko00360), *Soly105420* in phenylalanine, tyrosine, and tryptophan biosynthesis (ko00400), *Soly91330* in tyrosine metabolism (ko00350), and *Soly105420* in biosynthesis of amino acids (ko01230). Moreover, five genes related to chloroplast were identified: *Soly7100* in chlorophyll biosynthetic process (GO: 0015995), *Soly105920* encoding chloroplastic camphene/tricyclene synthase (*TPS3*), *Soly105420* encoding chloroplastic phospho-2-dehydro-3-deoxyheptonate aldolase 2 (*DHS2*), *Soly80150* encoding chloroplastic adenylate isopentenyltransferase 5 (*IPT5*), and *Soly85330* encoding chloroplastic MATE efflux family protein 2 (*DTX44*).

In the MEturquoise module (Figure 4B, Supplemental Table S2), seven genes were enriched in RNA-related pathways: *Soly9620*, *Soly9630*, *Soly11340*, and *Soly104560* in mRNA surveillance (ko03015); *Soly80810*, *Soly96870*, and *Soly112290* in aminoacyl-tRNA biosynthesis (ko00970); and *Soly104560* in RNA transport (ko03013). A number of genes involved in amino acid metabolism-related pathways were also identified: *Soly91190*, *Soly98550*, and *Soly104000* in the biosynthesis of amino acids (ko01230); *Soly74030* and *Soly104000* in cyanoamino acid metabolism (ko00460); *Soly98550* and *Soly104000* in glycine, serine, and threonine metabolism (ko00260); *Soly91190* and *Soly98550* in phenylalanine, tyrosine, and tryptophan biosynthesis (ko00400); and *Soly108800* in valine, leucine, and isoleucine degradation (ko00280) as well as beta-alanine metabolism (ko00410).

There were four genes associated with fatty acid metabolism in the MEturquoise module: *Soly5750* and *Soly6540* in linoleic acid and alpha-linolenic acid metabolism (ko00591, ko00592); *Soly90410* in fatty acid metabolism (ko01212) and fatty acid elongation (ko00062); and *Soly10860* in sphingolipid metabolism (ko00600). Additionally, three genes related to carbon metabolism were identified: *Soly104000* and *Soly108800* in carbon metabolism (ko01200); *Soly112290* in porphyrin and chlorophyll metabolism (ko00860); and *Soly104000* in one carbon pool by folate (ko00670). Furthermore, there were two genes related to sugar metabolism: *Soly90410* in pentose and glucuronate interconversions (ko00040) as well as fructose and mannose metabolism (ko00051); and *Soly74030* in starch and sucrose metabolism (ko00500) (Table S3).

### 2.3. Generation of Transgenic Plants

**Figure 5:**
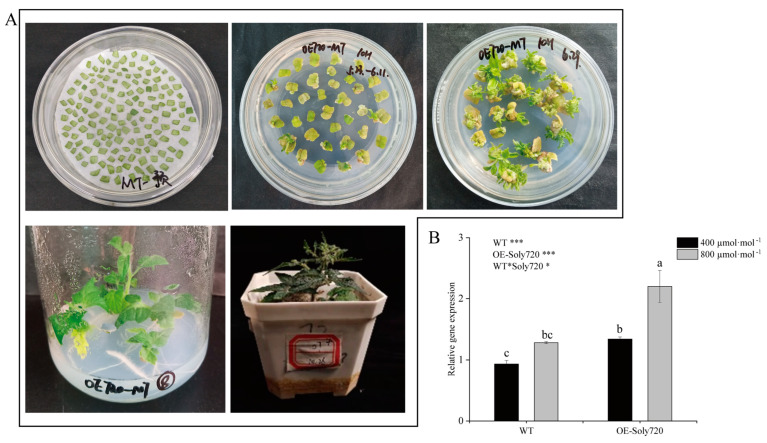
Generation of *Soly720*-transgenic tomato plants. (**A**) *Agrobacterium*-mediated tomato transformation. (**B**) Relative gene expression level of *Soly720* in transgenic (OE-Soly720) and wild-type (WT) plants. Data are the means ± standard deviations of three biological replicates. Different letters above the error bars indicate significant differences among the group means according to Tukey’s test (*p* < 0.05). *, *p* < 0.05; ***, *p* < 0.001; NS, not significant.

Based on previous research and gene functional annotations, the *Soly720* gene was selected to study its function in plant growth and development of tomato. The full-length coding sequence of *Soly720* was inserted into the pCAMBIA1305.1 vector containing the 35S promoter of cauliflower mosaic virus to construct a 35S::Soly720 overexpression vector. Transgenic plants overexpressing *Soly720* were obtained using *Agrobacterium*-mediated transformation (Figure 5A). Quantitative real-time PCR (qPCR) analysis revealed that compared to wild-type (WT) plants, the gene expression level of *Soly720* in transgenic plants was 1.44 and 1.71 times higher under natural and CO_2_-enriched conditions, respectively (Figure 5B).

### 2.4. Effects of Constitutive Overexpression of Soly720 on Plant Growth

**Figure 6:**
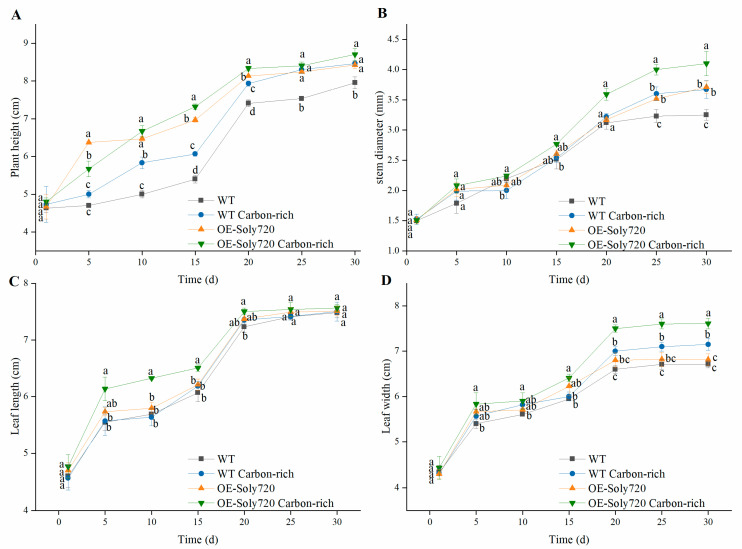
Morphological parameters of wild-type (WT) and *Soly720*-transgenic (OE-Soly720) tomato plants under natural and CO_2_-enriched conditions. (**A**) Average plant height; (**B**) average stem diameter; (**C**) average leaf length; (**D**) average leaf width. Different letters above the error bars indicate significant differences among the group means according to Tukey’s test (*p* < 0.05). Data are the means ± standard deviations of three biological replicates.

To assess the effect of overexpressing the *Soly720* gene on plant growth, the morphological parameters of transgenic tomato were measured. Under natural and CO_2_-enriched conditions, plant height, stem diameter, leaf length, and leaf width of T1-generation transgenic plants were all higher than those of WT (Figure 6). For example, under natural conditions, the average plant height of transgenic plants increased by 35.5%, 30.0%, 29.6%, 8.0%, 11.4%, and 5.9% compared to that of WT plants at days 5, 10, 15, 20, 25, and 30, respectively. Less prominent increases occurred in the corresponding average plant height of transgenic plants in the high-CO_2_ environment (13.4%, 15.5%, 20.0%, 5.0%, 1.2%, and 2.8%, respectively; Figure 6A).

Compared to that of WT plants, the average stem diameter of transgenic plants increased by 5.5–12.0% under high-CO_2_ conditions during the experimental period (Figure 6B). The average leaf length of transgenic plants also increased by 4.2–0.4% under natural conditions, with a further increase of 8.9–0.8% under high-CO_2_ conditions (Figure 6C). A similar pattern was observed in the average leaf width in transgenic plants, which increased by 5.0–1.6% under natural conditions and by 4.1–1.4% under high-CO_2_ conditions (Figure 6D).

### 2.5. Effects of Constitutive Overexpression of Soly720 Gene on Photosynthetic Characteristics

**Figure 7:**
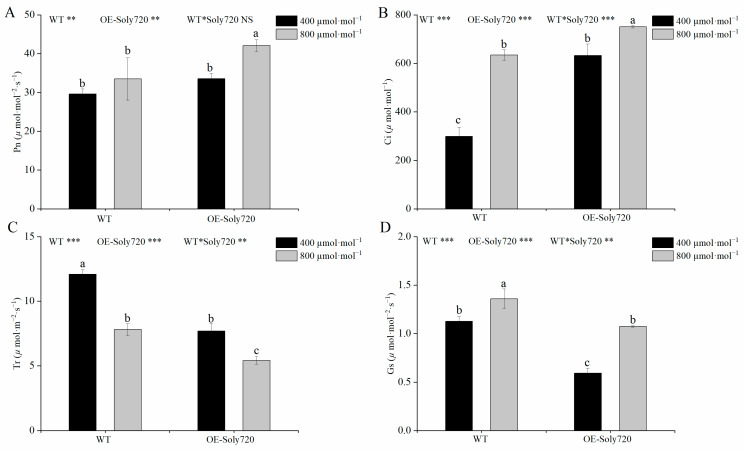
Photosynthetic characteristics in leaves of wild-type (WT) and *Soly720*-transgenic (OE-Soly720) tomato plants under different CO_2_ conditions. (**A**) Net photosynthetic rate (Pn); (**B**) intercellular CO_2_ concentration (Ci); (**C**) transpiration rate (Tr); and (**D**) stomatal conductance (Gs). Data are the means ± standard deviations (*n* = 3). Different letters above the error bars indicate significant differences among the group means according to Tukey’s test (*p* < 0.05). *, *p* < 0.05; **, *p* < 0.01; ***, *p* < 0.001; NS, not significant.

The effects of overexpressing the *Soly720* on transgenic tomato plants were further investigated by measuring leaf photosynthetic parameters. Under high-CO_2_ conditions, the net photosynthetic rate in transgenic plants was 1.26 times higher than that of WT plants (*p <* 0.05; Figure 7A). Compared to WT plants, the intercellular CO_2_ concentration in transgenic plants was 2.12 and 1.18 times higher under natural and high-CO_2_ conditions, respectively (*p <* 0.05; Figure 7B). In contrast, the transpiration rate (Figure 7C) and stomatal conductance (Figure 7D) in transgenic plants were significantly lower than those in WT plants, by 36% and 47% under natural conditions and by 31% and 21% under CO_2_-rich conditions, respectively.

**Figure 8:**
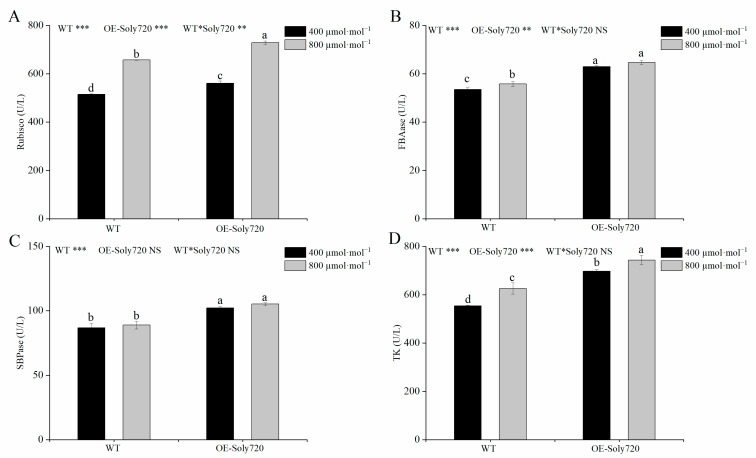
Key photosynthetic enzyme activities in leaves of wild-type (WT) and *Soly720*-transgenic (OE-Soly720) tomato plants under different CO_2_ conditions. (**A**) Ribulose-1, 5-bisphosphate carboxylase/oxygenase (RuBisCO) activity; (**B**) Fructose-1, 6-bisphosphate aldolase (FBAase) activity; (**C**) Transketolase (TK) activity; (**D**) Sedoheptulose-1,7-bisphosphatase (SBPase) activity. Data are the means ± standard deviations (*n* = 3). Different letters above the error bars indicate significant differences among the group means according to Tukey’s test (*p* < 0.05). *, *p* < 0.05; **, *p* < 0.01; ***, *p* < 0.001; NS, not significant.

**Figure 9:**
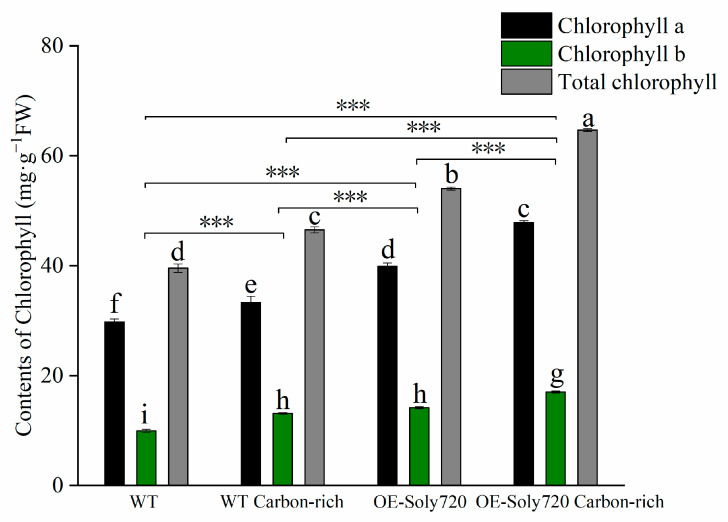
Contents of chlorophyll a, chlorophyll b, and total chlorophyll in leaves of wild-type (WT) and *Soly720*-transgenic (OE-Soly720) tomato plants under different CO_2_ conditions. Data are the means ± standard deviations (*n* = 3). Different letters above the error bars indicate significant differences among the group means according to Tukey’s test (*p* < 0.05). ***, *p* < 0.001.

The activities of key photosynthetic enzymes were also measured in tomato plants. The RuBisCO activity followed the following order: transgenic plants under CO_2_-enriched conditions> WT plants under CO_2_-enriched conditions> transgenic plants under natural conditions> WT plants under natural conditions (Figure 8A). The fructose-1, 6-bisphosphate aldolase (FBAase) and SBPase activities were significantly higher in transgenic plants than in WT plants, but no response to CO_2_ enrichment was observed (Figure 8B,D). The transketolase (TK) activity was also enhanced in transgenic plants compared with WT plants and responded positively to CO_2_ enrichment (Figure 8C). Moreover, the chlorophyll a, chlorophyll b, and total chlorophyll contents in tomato leaves were measured, which mirrored the pattern of TK activity (Figure 9).

## 3. Discussion

The elevation of atmospheric CO_2_ concentration has a fertilization effect on plant growth and development by affecting photosynthesis. Thus, high CO_2_ concentration is conducive to increasing plant biomass and the yield of crop product organs in greenhouses [22,23,24,25]. However, carbon uptake by plants is constrained as global temperatures and atmospheric CO_2_ concentrations continue to rise. The complicated strategies of CO_2_-enriched plants to utilize carbon sources have not been sufficiently studied. Here, we constructed gene co-expression networks in tomato plants under high-CO_2_ conditions using WGCNA. Then, we explored the hub genes and metabolic response pathways in the gene regulatory network of tomato in response to CO_2_ enrichment. In two highly correlated network modules (MEblack and MEturquoise), a number of genes were enriched in multiple RNA-related and amino acid metabolism pathways. This provides evidence that CO_2_ enrichment accelerates protein renewal in tomato plants.

It has been reported that CO_2_-enriched plants have increased membrane stability under high-temperature stress [26]. Fatty acids are the main chemical components of the phospholipid bilayer that maintains the stability of cell membranes. In the co-expression network of the MEturquoise module, a few genes were found enriched in fatty acid metabolism pathways. This indicates that tomato plants regulate cell membrane stability by modulating fatty acid metabolism under CO_2_ enrichment. Furthermore, the concentration of CO_2_ in the ambient environment has a direct impact on the levels of glucose, fructose, and overall reducing carbohydrates in plants [27]. Genes related to carbohydrate metabolism pathways were also identified in the co-expression network of the MEturquoise module, indicating that CO_2_ enrichment affects carbohydrate metabolism in tomato. Carbohydrate plays multiple roles in plants by regulating fruit quality and plant resistance.

To demonstrate the regulatory role of the chloroplast cell component-related gene *Soly720* in CO_2_-enriched tomato, we compared the morphological parameters of *Soly720*-transgenic and WT plants. Compared to WT plants, transgenic plants showed higher plant height, leaf length, leaf width, and stem diameter (by 2.8%, 0.8%, 6.4%, and 11.7%, respectively) under CO_2_ enrichment over the 30-day experimental period. The results suggest that overexpression of the *Soly720* gene promotes plant growth from a morphological perspective and enhances the growth performance of tomato under CO_2_ enrichment. Therefore, *Soly720* is likely to be a key gene involved in the regulation of plant growth and response mechanisms to CO_2_ enrichment.

Chlorophylls and carotenoids are part of the light harvesting system that captures light energy and drives photosynthetic electron transport [28]. Therefore, we measured the chlorophyll content and net photosynthetic rate in *Soly720*-transgenic tomato plants to better understand the potential gene function. The chlorophyll content of transgenic plants was higher than that of WT plants and increased in response to CO_2_ enrichment. Transgenic plants also exhibited a higher net photosynthetic rate under CO_2_-enriched conditions compared to the normal control. These findings indicate that the *Soly720* gene is directly involved in the regulation of photosynthesis and plays a greater role in the high-CO_2_ environment.

Leaf stomata are vital for plant communication with the external environment and serve as the primary passages for CO_2_ and water movement in and out of plants. Liang et al. [15] found that the stomatal conductance of plants in a high-CO_2_ environment was lower than that of plants grown under normal conditions. Moreover, drought-stressed plants in the high-CO_2_ environment maintained better water status and higher water use efficiency at both leaf and whole plant levels. In this study, *Soly720*-transgenic tomato plants exhibited a reduction in stomatal conductance compared to WT plants under both normal and CO_2_-enriched conditions. This provides molecular evidence that the *Soly720* gene plays a role in regulating stomatal conductance among tomato plants. According to Liang et al. [15], stomatal conductance is regulated by abscisic acid and ethylene biosynthesis pathways in CO_2_-enriched plants. It would be interesting to verify whether similar hormonal regulatory pathways exit in *Soly720*-transgenic tomato plants.

Elevated concentrations of CO_2_ mainly result in the acceleration of RuBisCO carboxylation in C3 plants while inhibiting oxygenase reaction. This process ultimately enhances net photosynthesis in plants [29]. SBPase, a key enzyme in the Calvin cycle, is mainly responsible for the regeneration of CO_2_ molecular receptor RuBP. Thus, SBPase plays a vital role in the circulation of carbon sources in the Calvin cycle [30]. FBAase and TK, which are also essential enzymes in the Calvin cycle, affect CO_2_ assimilation in plant leaves and the flow of carbon [31,32]. The enzyme activities of RuBisCO, SBPase, TK, and FBAase were all enhanced in transgenic tomato plants than in WT plants under different CO_2_ conditions. Accordingly, the *Soly720* gene participates in the Calvin cycle and the regulation of carbon flow in tomato. The findings indicate that *Soly720* is of significance in controlling photosynthesis in tomato plants under high-CO_2_ conditions. In addition, chlorophyll-related genes have been reported to play important roles in plant response to environmental stresses [33]. In tomato, Cai et al. [32] found that the expression level of the chlorophyll aldolase gene, *SlFBA4*, changes significantly under low-temperature stress and that MDA content is reduced in *SlFBA4* gene overexpression transgenic tomato plants. Chloroplasts are essential for their function in plants, and by regulating chloroplast-related genes they also help to repair damaged photosystems under conditions of high temperature and intense light stress [34]. Therefore, the study of chloroplast genes could help to improve the adaption of tomato plants in response to environmental changes. At present, the relevant role of the *Soly720* gene has not been reported, which will need further experiments to explore.

## 4. Materials and Methods

### 4.1. WGCNA and Identification of Hub Genes

WGCNA was performed using the R package v1.68 [35] to construct the gene co-expression network of tomato in response to CO_2_ enrichment. The input data were gene expression profiles obtained by RNA sequencing in a pervious study [21]. Network construction and module identification were carried out using the pickSoftThreshold function with default parameters. The co-expression network was constructed using the top 100 hub genes in each selected network module and visualized using Cytoscape v3.9.1 [36]. GO and KEGG enrichment analyses were performed as previously described [37].

### 4.2. Plasmid Construction and Plant Transformation

The full-length coding sequence of the *Soly720* gene (*Solyc01g007720*) was amplified from tomato leaf cDNA by PCR with appropriate primers (forward primer: 5′-ATGGATAGTTCAATGTGCTCAT-3′ and reverse primer: 5′-TTATTTTCCCATTGAGGCCGAA-3′). The amplified sequence was inserted into the *SMA*I and *SPE*I sites of binary vector pCAMBIA1300. Transgenic ‘Micro-Tom’ tomato plants were obtained by *Agrobacterium tumefacien*-mediated transformation with strain EHA105 [38]).

### 4.3. Detection of Transgenic Plants

DNA was extracted from leaves of T0-generation transgenic plants using the DNA secure plant kit (Tiangen Biotech, Beijing, China). The plant expression vector resistance gene *Hygromycin B* (*HygB*) was used for PCR detection with the primers HygB-F (5′-TAGCGAGAGCCTGACCTATT-3′) and HygB-R (5′-GATGTTGGCGACCTCGTATT-3′). Overexpression of the target gene *Soly720* in the T0 generation of transgenic plants was verified by PCR using a primer pair for the hygromycin resistance gene (*HYG*). Seeds collected from 13 transgenic lines and WT plants were germinated overnight in culture dishes at 28 °C in the dark. Seedlings were then grown in a growth chamber for 16 days (25 °C) and 8 h (18 °C). When two true leaves developed, the T1-generation transgenic seedlings were transferred into a standard glasshouse in the experimental field of Shanxi Agricultural University (Jinzhong, China).

### 4.4. RNA Extraction and qPCR Analysis

Total RNA was extracted from leaves of T1 transgenic plants using the RNAasy kit (Qiagen, Inc., Hilden, Germany) as per the manufacturer’s protocol. First-strand cDNA was synthesized using the PrimerScript RT Reagent Kit (TaKaRa, Beijing, China). qPCR was performed with the SYBR Premix *ExTag* on the Bio-Rad CFX96 (Bio-Rad, Shanghai, China), with the *SlActin* gene as internal control. The qPCR primers were designed using Primer Premier v6.0 (Palo Alto, CA, USA) (YG *Soly720*-F: 5′-CTTTCGATTCACTCGTGGGAT-3′; YG *Soly720*-R: 5′-GCCGAACCTAAACCTGTGCT-5′). Gene expression levels were calculated using the 2^−ΔΔCT^ method [39].

### 4.5. Plant Morphological Measurements

Transgenic and WT plants were grown in a grown chamber (28/16 °C, 16/8 h, day/night) with 60% relative humidity. Thirty-day-old seedlings were treated with different concentrations of CO_2_ (natural: 400 µmol·mol^–1^; CO_2_-enriched: 800 µmol·mol^–1^). Plant height, leaf length, leaf width, and stem diameter were measured on the 5th, 10th, 15th, 20th, 25th, and 30th days of CO_2_ treatment. Each morphological parameter was measured for three biological and three technical replicates.

### 4.6. Plant Physiological Measurements

The content of chlorophyll was determined by colorimetry after acetone extraction. The net photosynthetic rate, transpiration rate, intercellular CO_2_ concentration, and stomatal conductance were measured using a LI-6400 portable photosynthesometer (LI-COR Biosciences, Lincoln, NE, USA). The activities of key photosynthetic enzymes were assayed using the Solarbio kit (Beijing Solarbio Science & Technology Co., Ltd., Beijing, China). Briefly, leaf samples (0.1 g each) were homogenized with 1 mL of extraction buffer in ice and then centrifuged at 10,000× *g* and 4 °C for 10 min; the supernatants were collected to measure RuBisCO activity by reading the absorbance at the wavelength of 340 nm at 20 s and 5 min 20 s. For FBAase, leaf extracts were centrifuged at 8000× *g* and 4 °C for 10 min, and the supernatants were collected to read the absorbance at the wavelength of 340 nm at 10 s and 310 s after 5 min of water bath at 25 °C. Enzyme-linked immunosorbent assay was used to measure TK and SBPase activities at the wavelength of 450 nm, after 10 min of centrifugation of leaf extracts at 3000 and 5000× *g*, respectively. 

## 5. Conclusions

This study investigated the comprehensive cross-response mechanisms of greenhouse tomato to CO_2_ enrichment. WGCNA results showed that tomato plants are rich in amino acid metabolism in high-CO_2_ environments and maintain the stability of cellular structure through fatty acid metabolism pathways. The hub genes in co-expression networks were mainly involved in plant growth and development, such as cellular components and photosynthesis. Under CO_2_-enriched conditions, transgenic plants overexpressing the photosynthesis-related gene *Soly720* showed enhanced growth performance and carbon assimilation ability based on morphological, photosynthetic, and enzymatical observations. Thus, *Soly720* plays a vital role in the response of tomato plants to high CO_2_ conditions. The results of this study could be useful for the cultivation of new tomato varieties in the context of global climate change.

## Data Availability

Data is contained within the article and supplementary material.

## Supplementary Materials

The following supporting information can be downloaded at: https://www.mdpi.com/article/10.3390/ijms25020757/s1.

## References

[1] Li W., Han X., Zhang Y., Li Z. (2007). Effects of elevated CO_2_ concentration, irrigation and nitrogenous fertilizer application on the growth and yield of spring wheat in semi-arid areas. Agric. Water Manag..

[2] Kaiser E., Zhou D., Heuvelink E., Harbinson J., Morales A., Marcelis L.F.M. (2017). Elevated CO_2_ increases photosynthesis in fluctuating irradiance regardless of photosynthetic induction state. J. Exp. Bot..

[3] Vega-Mas I., Pérez-Delgado C.M., Marino D., Fuertes-Mendizábal T., González-Murua C., Márquez A.J., Betti M., Estavillo J.M., González-Moro M.B. (2017). Elevated CO_2_ induces root defensive mechanisms in tomato plants when dealing with ammonium toxicity. Plant Cell Physiol..

[4] Ha T., Zhang X., Li J., Gao Y. (2017). Effects of supply amounts and frequencies of nutrient solution on plant growth and fruit quality of highly sugary tomato. Acta Agric. Boreal.-Occident Sin..

[5] Zhang Y., Li W., Liu X., Wang J., Tang Z., Yu J. (2021). Effect of potassium application rate on growth physiology, yield and quality of tomato cultivated in facility substrate. Acta Bot. Boreal.-Occident. Sin..

[6] Tomato Genome Consortium (2012). The tomato genome sequence provides insights into fleshy fruit evolution. Nature.

[7] Becklin K.M., Walker S.M., Way D.A., Ward J.K. (2017). CO_2_ studies remain key to understanding a future world. New Phytol..

[8] Pan C., Zhang H., Ma Q., Fan F., Fu R., Ahammed G.J., Yu J., Shi K. (2019). Role of ethylene biosynthesis and signaling in elevated CO_2_-induced heat stress response in tomato. Planta.

[9] Mamatha H., Rao N.K.S., Srinivasarao N.K., Laxman R.H., Bhatt R.M., Pavithra K.C. (2014). Impact of elevated CO_2_ on growth, physiology, yield, and quality of tomato (*Lycopersicon esculentum* Mill) cv. Arka Ashish. Photosynthetica.

[10] McGrath J.M., Lobell D.B. (2013). Reduction of transpiration and altered nutrient allocation contribute to nutrient decline of crops grown in elevated CO_2_ concentrations. Plant Cell Environ..

[11] Bencze S., Keresztényi I., Varga B., Kőszegi B., Balla K., Gémesné-Juhász A., Veisz O. (2011). Effect of CO_2_ enrichment on canopy photosynthesis, water use efficiency and early development of tomato and pepper hybrids. Acta Agronomica Hungarica..

[12] Drake B.G., Gonzalez-Meler M.A., Long S.P. (1997). More efficient plants: A consequence of rising atmospheric CO_2_. Annu. Rev. Plant Physiol. Plant Mol. Biol..

[13] Feng L., Wang K., Li Y., Tan Y., Kong J., Li H., Li Y., Zhu Y. (2007). Overexpression of SBPase enhances photosynthesis against high temperature stress in transgenic rice plants. Plant Cell Rep..

[14] Hasan M.K., Xing Q.F., Zhou C.Y., Wang K.X., Xu T., Yang P., Qi Z.Y., Shao S.J., Ahammed G.J., Zhou J. (2023). Melatonin mediates elevated carbon dioxide-induced photosynthesis and thermotolerance in tomato. J. Pineal Res..

[15] Liang K., Chen X., Liu F. (2023). Antagonistic or Compensatory: Crosstalk between ABA and Ethylene in Regulating Stomatal Behavior under High CO_2_ and Progressive Soil Drying. J. Exp. Bot..

[16] Rosales R., Romero I., Fernandez-Caballero C., Escribano M.I., Merodio C., Sanchez-Ballesta M.T. (2016). Low temperature and short-term high-CO_2_ treatment in postharvest storage of table grapes at two maturity stages: Effects on transcriptome profiling. Front. Plant Sci..

[17] Kolbe A.R., Studer A.J., Cornejo O.E., Cousins A.B. (2019). Insights from transcriptome profiling on the non-photosynthetic and stomatal signaling response of maize carbonic an hydrase mutants to low CO_2_. BMC Genom..

[18] Chen T., Wu H., Wu J., Fan X., Li X., Lin Y. (2017). Absence of Oβsca1 causes a CO_2_ deficit and affects leaf photosynthesis and the stomatal response to CO_2_ in rice. Plant J..

[19] Suzuki Y., Ishiyama K., Sugawara M., Suzuki Y., Kondo E., Takegahara-Tamakawa Y., Yoon D.K., Suganami M., Wada S., Miyake C. (2021). Overproduction of Chloroplast Glyceraldehyde-3-Phosphate Dehydrogenase Improves Photosynthesis Slightly under Elevated [CO_2_] Conditions in Rice. Plant Cell Physiol..

[20] Umnajkitikorn K., Sade N., Rubio Wilhelmi M.D.M., Gilbert M.E., Blumwald E. (2020). Silencing of OsCV (*chloroplast vesiculation*) maintained photorespiration and N assimilation in rice plants grown under elevated CO_2_. Plant Cell Environ..

[21] Zheng S., Chen Z., Nie H., Sun S., Zhou D., Wang T., Zhai X., Liu T., Xing G., Li M. (2020). Identification of differentially expressed photosynthesis- and sugar synthesis-related genes in tomato (*Solanum lycopersicum*) plants grown under different CO_2_ concentrations. Biotechnol. Biotechnol. Equip..

[22] Zhang Q., Zhu Z. (2015). The Effects of CO_2_ Enrichment on Physiological Mechanismsin Cucumber under Salt Stress. Tillage Cultiv..

[23] Kumari S., Agrawal M. (2014). Growth, yield and quality attributes of a tropical potato variety (*Solanum tuberosum*, L. cv *Kufri chandramukhi*) under ambient and elevated carbon dioxide and ozone and their interactions. Ecotoxicol. Environ. Saf..

[24] Schjørring J.K. (2013). Effects of elevated atmospheric CO2 on physiology and yield of wheat (*Triticum aestivum L*): A meta-analytic test of current hypotheses. Agric. Ecosyst. Environ..

[25] Wei Z., Du T., Li X., Fang L., Liu F. (2018). Interactive effects of elevated CO_2_ and N fertilization on yield and quality of tomato grown under reduced irrigation regimes. Front. Plant Sci..

[26] Yu J.J., Du H.M., Xu M., Huang B.R. (2012). Metabolic responses to heat stress under elevated atmospheric CO_2_ concentration in a cool-season grass species. J. Am. Soc. Hortic. Sci..

[27] Högy P., Fangmeier A. (2009). Atmospheric CO_2_ enrichment affects potatoes: 2. Tuber quality traits. Eur. J. Agron..

[28] Scheer H. (2013). Chlorophylls and Carotenoids. Encyclopedia of Biological Chemistry.

[29] Stitt M., Krapp A. (1999). The interaction between elevated carbon dioxide and nitrogen nutrition: The physiological and molecular background. Plant Cell Environ..

[30] Wang M.L. (2011). Molecular Cloning and Transformation of Sedoheptulose-1,7-bisphosphatase in *Lycopersicon esculentum*. Ph.D. Thesis.

[31] Yang Y., Xie J., Li J., Zhang J., Zhang X., Yao Y., Wang C., Niu T., Bakpa E.P. (2022). Trehalose alleviates salt tolerance by improving photosynthetic performance and maintaining mineral ion homeostasis in tomato plants. Front. Plant Sci..

[32] Cai B., Ning Y., Li Q., Li Q., Ai X. (2022). Effects of the Chloroplast Fructose-1,6-Bisphosphate Aldolase Gene on Growth and Low-Temperature Tolerance of Tomato. Int. J. Mol. Sci..

[33] Zhang Y., Zhang A., Li X., Lu C. (2020). The role of chloroplast gene expression in plant responses to environmental stress. Int. J. Mol. Sci..

[34] Zhang Y., Tian L., Lu C. (2023). Chloroplast gene expression: Recent advances and perspectives. Plant Commun..

[35] Langfelder P., Horvath S. (2008). WGCNA: An R package for weighted correlation network analysis. BMC Bioinform..

[36] Shannon P., Markiel A., Ozier O., Baliga N.S., Wang J.T., Ramage D., Amin N., Schwikowski B., Ideker T. (2003). Cytoscape: A software environment for integrated models of biomolecular interaction networks. Genome Res..

[37] Fuyou F., Zhang W., Li Y.Y., Wang H.L. (2017). Establishment of the model system between phytochemicals and gene expression profiles in Macrosclereid cells of Medicago truncatula. Sci. Rep..

[38] Kesiraju K., Mishra P., Bajpai A., Sharma M., Rao U., Sreevathsa R. (2020). Agrobacterium tumefaciens-mediated in planta transforma-tion strategy for development of transgenics in cotton (*Gossypium hirsutum* L.) with GFP as a visual marker. Physiol. Mol. Biol. Plants.

[39] Livak K.J., Schmittgen T.D. (2001). Analysis of relative gene expression data using real-time quantitative PCR and the 2−∆∆CT method. Methods.

